# Nerve Growth Factor Serum Levels Are Associated With Regional Gray Matter Volume Differences in Schizophrenia Patients

**DOI:** 10.3389/fpsyt.2019.00275

**Published:** 2019-04-26

**Authors:** Kristina Neugebauer, Christine Hammans, Tobias Wensing, Vinod Kumar, Wolfgang Grodd, Lea Mevissen, Melanie A. Sternkopf, Ana Novakovic, Ted Abel, Ute Habel, Thomas Nickl-Jockschat

**Affiliations:** ^1^Department of Psychiatry, Psychotherapy and Psychosomatics, Medical Faculty, RWTH Aachen University, Aachen, Germany; ^2^Jülich-Aachen Research Alliance, Jülich, Germany; ^3^Max-Planck-Institute for Biological Cybernetics, Tübingen, Germany; ^4^Iowa Neuroscience Institute, University of Iowa, Iowa City, IA, United States; ^5^Department of Psychiatry, Carver College of Medicine, University of Iowa, Iowa City, IA, United States

**Keywords:** schizophrenia, nerve growth factor, voxel-based morphometry, neuroimaging, functional decoding

## Abstract

Numerous neuroimaging studies have revealed structural brain abnormalities in schizophrenia patients. There is emerging evidence that dysfunctional nerve growth factor (NGF) signaling may contribute to structural brain alterations found in these patients. In this pilot study, we investigated whether there was a correlation between NGF serum levels and gray matter volume (GMV) in schizophrenia patients. Further, we investigated whether there was an overlap between the correlative findings and cross-sectional GMV differences between schizophrenia patients (n = 18) and healthy controls (n = 19). Serum NGF was significantly correlated to GMV in the left prefrontal lobe, the left midcingulate cortex, and the brainstem in schizophrenia patients. However, we did not find any correlations of NGF serum levels with GMV in healthy controls. Schizophrenia patients showed smaller GMV than healthy controls in brain regions located in the bilateral limbic system, bilateral parietal lobe, bilateral insula, bilateral primary auditory cortex, left frontal lobe, and bilateral occipital regions. In a conjunction analysis, GMV in the left midcingulate cortex (MCC) appears negatively correlated to NGF serum levels in the group of schizophrenia patients and also to be reduced compared to healthy controls. These results suggest an increased vulnerability of schizophrenia patients to changes in NGF levels compared to healthy controls and support a role for NGF signaling in the pathophysiology of schizophrenia. As our pilot study is exploratory in nature, further studies enrolling larger sample sizes will be needed to further corroborate our findings and to investigate the influence of additional covariates.

## Introduction

Structural brain abnormalities in schizophrenia patients have been repeatedly demonstrated by neuroimaging studies and were robustly confirmed by numerous meta-analyses (1–8), supporting the hypothesis that schizophrenia is associated with altered cerebral connectivity and morphology (9–11). The most consistent gray matter abnormalities were found in fronto-temporo-thalamic regions lateralized to the dominant hemisphere (3, 5, 6, 8, 12, 13). Notably, these changes were reported to be evident in first episode patients (14–18) and to be more pronounced with increased duration of the illness and earlier age of onset (2–3, 4, 6, 8, 12, 17, 19–24). Gray matter reductions were also frequently reported—although with lesser consistency—for cerebellar (21, 25), occipital (26, 27), and parietal (1, 4, 28) structures.

Anatomically, decreased amounts of cortical neuropil, deficits in dendritic arborization, and cortical afferents were reported to be contributing volume reductions in schizophrenia (29–33). In some brain areas, gray matter volume (GMV) reduction partially can be explained by a decrease in synaptic conjunctions (34, 35). This relation was investigated particularly for the dorsolateral prefrontal cortex and suggests influence on cognitive functions in schizophrenia patients (34, 36).

It seems likely that the etiopathogenesis of the structural differences found in schizophrenia patients is multifactorial and related to different interacting pathophysiological mechanisms (37). Members of the neurotrophin protein family, including nerve growth factor (NGF), have been repeatedly implicated not only in the pathogenesis of schizophrenia *per se*, but are plausible candidates to be involved also in the development of these neuroanatomical anomalies (38–40). Consequently, neurotrophins with their crucial role of contributing to the survival of neurons and the reorganization of neural circuits appear as worthwhile candidates to be studied in brain structural changes in schizophrenia.

NGF is essentially involved in neural development, differentiation, and maintenance of neurons not only in development, but throughout life, and has both short-time and long-term effects on brain structure and function by inducing neurite outgrowth as well as the length and complexity of dendritic length in cortical neurons (38, 39, 41–45). Although only few studies have investigated correlations between NGF levels in the peripheral blood and the central nervous system, several lines of evidence suggest that peripheral NGF serum levels allow valid conclusions on central nervous concentrations. Animal studies have shown that NGF penetrates the blood–brain barrier (46, 47) significantly better than albumin or IgG (48). In particular, NGF has been pointed out as the neurotrophin with the fastest passage of the blood–brain barrier in a mouse model (49). These properties render peripheral NGF as one of the most promising candidates within the neurotrophin family to infer on central levels. Recent evidence points to a reliable correlation between peripheral and central nervous NGF levels in humans, at least in healthy controls (50). Although further studies on larger collectives are warranted to corroborate these findings, we regard this as a highly encouraging lead that findings from animal models might be translatable to humans. Moreover, several studies have reported associations between peripheral NGF levels and various aspects of schizophrenia pathophysiology. For example, in schizophrenia patients, peripheral NGF levels were reported to be correlated with abnormal electrophysiological measures, namely p300 (51), while antipsychotic treatment was associated with an increase in NGF serum levels (24). Considering these findings, NGF serum levels may be a highly promising marker for central NGF concentrations.

NGF dysfunction, either due to pathological alteration of its concentration or its action on the receptor may contribute to impaired neuronal development, neuroplasticity, and synaptic disconnections. These changes may underlie structural and functional alterations observed in schizophrenia (52–54).

NGF serum levels in schizophrenia patients have been repeatedly reported to be significantly reduced when compared to healthy subjects (54, 55). Besides decreased NGF levels in the peripheral blood, decreased levels of NGF in the cerebrospinal fluid (CSF) (50) and decreased levels of NGF receptors (56), elevated NGF autoantibodies (57), and altered NGF gene expression (56) have been observed. Thus, changes of NGF levels might be involved in the formation of structural brain alternation of schizophrenia (24, 53).

While studies actually are still scarce, pioneer studies suggest a putative influence of neurotrophins on neural structure in schizophrenia patients. For example, a correlation was found between NGF autoantibodies in serum and total volume of the frontal lobe in schizophrenia patients (58). Another study explored the impact of the BDNF Val66Met gene variant in schizophrenia. This single nucleotide polymorphism (SNP) maps to the neurotrophin precursor protein of BDNF (brain-derived neurotrophin factor) (proBDNF) and is correlated with BDNF serum levels (59). Val66Met genotype was shown to correlate with reduced GMV in temporal and occipital regions known to be associated with verbal memory and visuospatial abilities in schizophrenia patients as well as healthy subjects (60). Another study suggests a contribution of the age-related decline of BDNF serum levels to hippocampal volume as well as memory decline in late adulthood (61). In patients with systemic lupus erythematosus with neuropsychiatric manifestation, immune cell NGF levels were found to be correlated with brain atrophy reflected by lateral ventricle enlargement and thalamic volume loss (62).

This study aimed to investigate a potential correlation between NGF serum levels and brain structural differences in schizophrenia. Furthermore, a potential overlap between the results of a whole brain correlation analysis between GMV and NGF serum levels and the results of GMV reductions in schizophrenia patients were identified in a conjunction analysis.

## Materials and Methods

### Subjects

Eighteen schizophrenia patients from the Department of Psychiatry, Psychotherapy and Psychosomatics, University Hospital RWTH Aachen, Aachen, Germany, and 19 gender- and age-matched healthy controls from the general population participated in the present study (**Table 1**). All subjects were of Caucasian descent and right-handed. Diagnosis of schizophrenia was established using the *International statistical classification of diseases and related health problems (ICD-10)* diagnostic criteria (code F20.X). Symptom severity in schizophrenia patients was measured using the Positive and Negative Syndrome Scale (PANSS) (63). Exclusion criteria for all subjects were 1) current substance or alcohol abuse or any diagnosed substance abuse or addiction in the past (*ICD-10*: F10.1 or .2 - 19.1 or .2), 2) severe medical conditions such as chronic or acute diseases (i.e., infections, allergies), 3) general contraindications to MRI scanning, 4) gross morphological changes on MRI such as cerebral atrophy, hydrocephalus, or previous injury, and 5) incomplete scanning. Subjects treated with benzodiazepines were not included, except the drug was at least in its second elimination half-life period after intake. Additional exclusion criteria for the healthy control subjects included any personal or family history of psychiatric disorders in first-degree relatives. The study protocol was approved by the local ethics committee of the RWTH Aachen University. Before being enrolled in the study, written informed consent was obtained from each subject in accordance with the Declaration of Helsinki.

**Table 1:** Demographic and clinical characteristics of the study subjects.

	SZ	HC	Statistics
N	18	19	
*Demographics*			
Age (years)	36.94 ± 9.90	35.79 ± 11.56	t = −.326, p = .747
Gender (female/male)	7/11	7/12	χ^2^ = .016, p = .898
Education (years)	16.17 ± 4.32	18.79 ± 3.91	t = 1.939, p = .061
BMI	27.08 ± 4.91	25.04 ± 5.05	z = −1.383, p = .169
*Biophysiology*			
Smokers/ex−smokers	9/0	1/1	
Nicotine (pack-years)	10.61 ± 15.97	4.63 ± 14.64	z = −1.853, p = .061
Sports (h/week)	1.75 ± 1.99	4.34 ± 3.5	z = 2.825, p = .004
NGF (pg/ml)	.0546 ± .005	.0574 ± .007	z = −1.401, p = .169
*Clinical*			
Duration illness (years)	12.58 ± 9.67		
PANSS			
Positive	15.33 ± 6.17		
Negative	23.61 ± 9.56		
General	43.83 ± 15.77		
Total	84.78 ± 29.13		
*Medication*			
OLZ (mg/day)	22.14 ± 11.94		
Atypicals (%)	100%		
Atypicals mono	56%		
Atypicals combi	33%		
Atypical plus typical	11%		

SZ, schizophrenia patients; HC, healthy controls; n, number of subjects per group; BMI, body mass index; pg, pictogram; OLZ, olanzapine equivalent; atypical, atypical antipsychotic; mono, monotherapy; combi, combi-therapy; typical, typical antipsychotic. Values are mean ± SD.

### Clinical Measures

We systematically assessed clinical variables, such as duration of disease and medication, by a detailed medical interview. Also, lifestyle habits such as smoking and weekly amount of physical activity were assessed, considering that these factors may influence the outcome of the disease and the neurotrophin levels (64–67). For antipsychotic medication, equivalent dose of olanzapine was calculated by using the conversion scale proposed by Gardner and colleagues (68). As antipsychotic medication (24) and body mass index (BMI) (69) have been shown to exert an influence on NGF serum levels, we explored whether these parameters were correlated in the entire sample, and the schizophrenia patients, as well as the healthy controls alone.

### NGF Serum Level Assessment and Analysis

Subjects were allowed to have their usual breakfast in the morning. A venous blood sample of 10 ml was collected in serum tubes from all subjects at 8:00 AM to control for circadian changes in NGF levels (70). Subsequently, serum was obtained after centrifugation at 2,000 rpm for 10 min at room temperature and then stored at −80°C in polypropylene tubes until analysis to avoid adhesion of NGF to the tube surface (71). Mature/biological active beta-NGF serum levels were measured with a double antibody sandwich enzyme-linked immunosorbent assay (ELISA) using a commercial kit (Human NGF/NGF Beta PicoKine™ ELISA Kit, Boster Biological Technology Co., Inc., USA) according to the manufacturer’s instructions^1^. The assay has a sensitivity of <1 pg/ml; the assay range comprises 15.6–1,000 pg/ml. For each sample, standard and blank control were carried out in duplicate by trained research personnel and the results were averaged.

### Magnetic Resonance Imaging Data Acquisition and Processing

Imaging was performed immediately after the acquisition of the individual blood samples on a Siemens Trio 3T MRI scanner (Siemens Medical Systems, Erlangen, Germany) using a 32-channel head coil. A T1-weighed magnetization prepared rapid acquisition gradient echo (MP-RAGE) sequence that was used to acquire anatomical images covering the whole brain [echo time (TE) = 2.52 ms, repetition time (TR) = 1,900 ms, inversion time (TI) = 900 ms, flip angle = 9°, resolution matrix = 256 × 256, slices = 176, slice thickness = 1 mm, voxel size = 0.976 × 0.976 × 1 mm, duration = 7:49 min]. All images were collected during a single imaging session. Whole brain voxel-based morphometry (VBM) (72) analysis was carried out on the T1-weighted images using the Diffeomorphic Anatomical Registration Through Exponentiated Lie Algebra (DARTEL) segmentation algorithm (73) as implied in SPM12. Image intensity inhomogeneity correction (bias-field correction) was achieved using SPM bias correction algorithm implemented in SPM segment (72). Reorientation of the images was fixed to the anterior commissure. The automatic detection of different tissue types within the T1-weighted images was archived using the standard segmentation model in SPM (72). The resulting gray matter ribbon was used for achieving accurate intersubject alignment using DARTEL (73). After the iterative alignment process (74), DARTEL generated flow field images as well as the DARTEL template. The resulting flow field maps and the DARTEL template were used to create the smoothed [6-mm full width at half maximum (FWHM)], Jacobian scaled, spatially normalized gray matter images into the Montreal Neurological Institute (MNI) coordinate system through nonlinear transformation (74, 75) before calculation of GMV and group comparison. With this choice of parameters, an affine transformation leads to a correction for differences in whole-brain volumes. Hence, our approach picks up *relative regional*, not *absolute*, brain volumes.

### Correlation Between Gray Matter Volume and Nerve Growth Factor Serum Levels

Whole-brain correlation analysis between NGF serum levels and GMV were performed for schizophrenia patients and healthy controls separately, as well as a combined group of patients and controls to identify disease-specific associations between NGF and GMV. The main analyses were performed including voxel-wise GMV values as the dependent variable and NGF serum levels as a predictor. Group status was included as an additional regressor in the general sample (i.e., patients and healthy controls combined).

For all tests general linear model (GLM) was used to determine the statistical inference. The cluster level multiple comparison correction was performed to delineate the significant clusters (at p < .001, uncorrected). To focus all subsequent analyses on clusters of biological meaning, we chose a cluster extent threshold of k > 100 voxels. As brain structural changes after disease onset show a regional pattern, we purposefully chose this approach that is especially prone to pick up these changes.

### Overlap of Correlation Analysis and Group Comparison

To identify possible overlaps between clusters correlated to NGF levels and GMV differences, a conjunction analysis was conducted.

First, to achieve a whole brain voxel-wise group GMV comparison between patients and controls, GLM analysis was performed using a two-sample t-test (p < .001) to identify core structural gray matter differences in schizophrenia patients. Cluster-level threshold was set at p < .05 using family-wise error correction (FWE) with a voxel-level threshold set at p < .001. To focus all subsequent analyses on clusters of biological meaning, we chose a cluster extent threshold of k >100 voxels.

To objectify a potential anatomical overlap, we computed a conjunction analysis between the results of the group comparison (schizophrenia patients and healthy controls) and the findings of our correlation analyses (correlation between serum NGF levels and GMV). That is, by computing the intersection of the thresholded maps from the group comparison and the correlation analyses (76), we aimed to investigate an overlap between structural changes associated with schizophrenia and GMVs correlated with NGF.

### Anatomical Labeling

We used the SPM Anatomy Toolbox 2.2c (77–79) to obtain an anatomical localization of the resulted clusters from the group comparisons as well as the correlation analyses. The Anatomy Toolbox provides two different sets of information for a given cluster: a cortical area and a probabilistic microanatomical allocation based on cytoarchitectural probabilistic mapping (77–79). Wherever obtainable, we report both the macro- and the microanatomical labels. In all other cases, we report the macroanatomical allocations as provided by the Anatomy Toolbox.

### Functional Decoding

To obtain an observer-independent data-driven functional characterization of the clusters ensuing from our VBM analyses, we assessed whether we could find significant associations with behavioral domains and paradigm classes provided by the BrainMap data base (http://www.brainmap.org) (80, 81), a data base of published functional neuroimaging experiments. Here, behavioral domains describe the mental process likely to be isolated by an experimental contrast. They are divided into main categories: action, cognition, emotion, interception and perception, and their related subcategories. Furthermore, paradigm classes representing specific and established tasks of the experiments were used to determine activation in defined brain regions (for a detailed BrainMap taxonomy, see http://www.brainmap.org/taxonomy). Since it was our aim to characterize the physiological function of the identified brain regions, we only referred to studies on healthy subjects. Forward and reverse inference was performed. The forward inference approach denotes the probability of a particular mental process activating a specific brain region [P(activation|task)] in comparison to the probability of (random) activation in this specific brain region at baseline [P(activation)]. In the interest of clarity, results for forward and reverse inference are displayed in one column. Significance was assessed using a binominal test (p < .05), corrected for multiple comparisons using false discovery rate (FDR). The reverse inference approach attempts to identify the most likely psychological process, described in the categories of behavioral domains and paradigm classes, being implemented given neural activation in a specific brain region or network [P(task|activation)]. Significance was assessed here using a chi-square test (p < .05), FDR-corrected.

## Results

### Correlation of Clinical Data and Gray Matter Volume With Nerve Growth Factor Serum Levels

**Figure 1:**
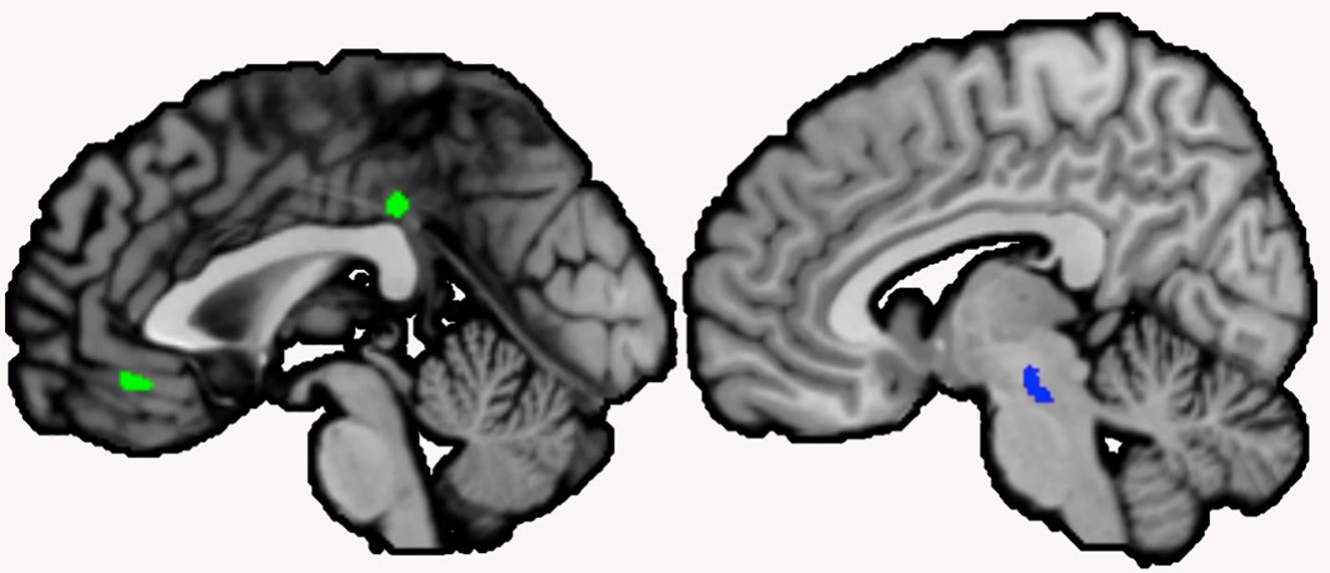
Clusters correlated with serum nerve growth factor (NGF) in schizophrenia patients. *Note:* Clusters correlated with serum NGF levels in schizophrenia patients were located in the left midcingulate cortex (extending over the midline) and the left mid orbital gyrus (extending over the midline; negative correlation, shown in green), and the brainstem (positive correlation, shown in blue; sagittal view, *p* < .001, cluster-level threshold *p* < .05, FWE-corrected, *k* > 100)

In schizophrenia patients, serum NGF levels were found to be positively correlated with GMV in the pons (**Table 2**, **Table S1** and **Figure 1**). The cluster was significantly associated with the paradigm class of pain monitoring and pain discrimination. Two clusters were negatively correlated between NGF serum levels and GMV, one cluster located in the left mid orbital gyrus extending into the right hemisphere, and the other in the left MCC (**Table 2**, **Figure 1**). Both clusters were found to be associated with reward related tasks.

**Table 2:** Results of VBM analyses: Correlation analysis.

Cluster	k (voxels)	MNI coordinates			Macroanatomy	Assigned cytoarchitecture
		x	y	z		
Positive correlation of GMV with NGF in SZ patients						
Cluster 1	133	−8	−20	−12	R brainstem	R unknown area
		−6	−23	−21	R brainstem	R unknown area
Negative correlation of GMV with NGF in SZ patients						
Cluster 1	230	−3	37	−12	L mid orbital gyrus	L area s32
Cluster 2	111	0	−32	35	L midcingulate cortex	

Whole-brain correlation analysis between NGF serum levels and GMV were performed to identify disease-specific associations between NGF and GMV. No cluster was found to be significantly negatively or positively correlated with NGF in either the healthy control or the combined group of patients and controls. NGF, nerve growth factor; GMV, gray matter volume.

For detailed information on assigned probabilistic cytoarchitectonic areas, please refer to area s32 (82).

We did not find any cluster to be significantly negatively or positively correlated with NGF in either the healthy control or the combined group.

NGF serum levels did not differ significantly between schizophrenia patients and healthy controls (z = −1.401, p = .169). However, NGF levels tended to be reduced in schizophrenia patients. NGF was found to be symmetrically distributed in healthy controls (p = .076), however, not symmetrically distributed in patients (p = .007).

In our study sample, no significant correlation was found between BMI and NGF serum levels in patients, as well as in healthy controls (SZ: r_s_ = 0.251, p = 0.316, HC: r_s_ = −0.418, p = 0.075). In schizophrenia patients, no significant correlation was found between daily olanzapine (equivalent) dose and NGF serum levels (r_s_ = 0.87, p = 0.732). In our study sample, significant correlation was not found between sex, BMI, years of education, and pack-years nor between the amount of weekly physical exercise and NGF serum levels in patients as well as in healthy controls. No correlation between age and NGF in patients was found, however, in healthy controls (rs = −.507, p = .027). Years of education were significantly correlated to NGF serum levels in controls (rs = −.697, p = .001), but not in schizophrenia patients. In schizophrenia patients, no correlation between neither duration of disease, daily olanzapine (equivalent) dose, or neither the results of PANSS test (positive, negative, general, and total scale) and NGF serum levels were found.

### Group Comparison and Conjunction Analysis

**Figure 2:**
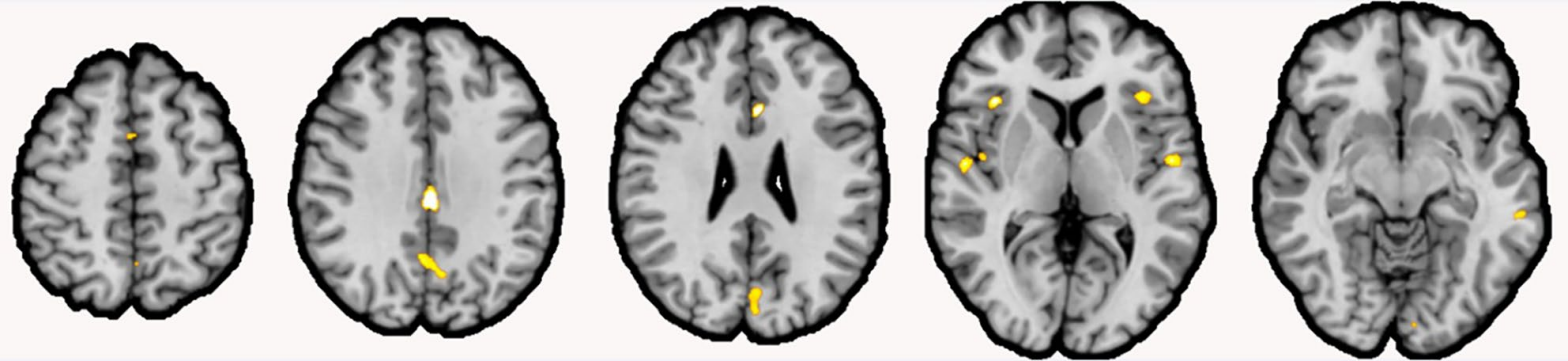
Gray matter volume (GMV) reductions in schizophrenia patients compared to healthy controls. *Note:* Clusters of reduced GMV in schizophrenia patients (shown in yellow) compared to healthy controls were located inter alia the bilateral insula, the left Rolandic operculum, the left area TE 1.2 and the right area TE 1.0, the right anterior cingulate cortex (ACC; extending over the midline), left posterior–medial frontal gyrus, the left midcingulate cortex (MCC; extending over the midline), the right precuneus (extending over the midline), and the visual areas hOc1 and hOc2 (both extending over the midline; coronal view, *p* < .001, cluster-level threshold *p* < .05, FWE-corrected, *k* > 100).

We found significant reduction of GMV in schizophrenia patients in 11 clusters. Clusters were located in the cingulate [left midcingulate cortex (MCC), right anterior cingulate cortex], bilaterally parietal lobes, bilateral insula, bilateral auditory cortex superior temporal lobe, left frontal lobe, and the bilateral occipital lobe (**Table 3**, **Figure 2**).

**Table 3:** Results of VBM analyses: Clusters of reduced GMV in SZ patients.

Cluster	k (voxels)	MNI coordinates			Macroanatomy	Assigned cytoarchitecture
		x	y	z		
Cluster 1	2,618	5	−77	15	R calcarine gyrus	R area hOc2 (V2)
		−2	−85	22	L cuneus	L area hOc3d
		2	−85	20	L cuneus	L area hOc2 (V2)
		−2	−84	13	L calcarine gyrus	L area hOc2 (V2)
		−1	−85	14	L calcarine gyrus	L area hOc2 (V2)
						
Cluster 2	718	1	−28	33	L midcingulate cortex	
Cluster 3	580	7	−65	31	R precuneus	
		−4	−57	35	L precuneus	
		−3	−59	33	L precuneus	
		3	−62	31	L precuneus	
		1	−61	32	L precuneus	
Cluster 4	371	4	−90	−6	L calcarine gyrus	L area hOc1 (V1)
Cluster 5	297	−39	22	1	L insula lobe	
Cluster 6	264	53	−8	4	R heschls gyrus	R area TE 1.0
Cluster 7	257	37	25	2	R insula lobe	
Cluster 8	256	4	18	25	R anterior cingulate cortex	R area 33
Cluster 9	246	−44	−1	0	Left Rolandic operculum	
		−43	−6	1	Left insula lobe	
		−48	1	0	Left superior temporal gyrus	
Cluster 10	241	0	4	48	Left post.-med. frontal gyrus	
Cluster 11	149	−54	−9	4	Left superior temporal gyrus	L area TE 1.2

L, left; R, right, V1, visual area 1; V2, visual area 2; Post.-Med., posterior–medial. For detailed information on assigned probabilistic cytoarchitectonic areas, please refer to area hOc1 and area hOc2 (83); area hOc3a (84); area TE 1.0 and area TE 1.2 (85, 86); area 33 (82).

The clusters showing reduced GMV in schizophrenia patients were associated with social cognition, reasoning, action execution and language-related processes (see **Table 5** for an overview).

**Table 4:** Results of VBM analyses: Conjunction analysis.

Cluster	k (voxels)	MNI coordinates			Macroanatomy	Assigned cytoarchitecture
		x	y	z		
Cluster 1	60	0	−31	34	L midcingulate cortex	

Conjunction analysis revealed an overlap between the NGF serum level to GMV correlation and the GMV group comparison in the left MCC in schizophrenia patients. L, left.

**Table 5:** Functional characterization for results of group difference and correlation analyses.

Cluster	Region	Behavioral domain *Mental process*	Paradigm class *Experimental task*
Positive correlation of GMV with NGF in SZ patients			
Cluster 1	Brainstem	No significant effects.	Pain monitor/discrimination
Negative correlation of GMV with NGF in SZ patients			
Cluster 1	Area s32	Emotion	Reward
		Cognition	
Cluster 2	L MCC	Emotion	Reward
		Cognition	
Clusters of reduced GMV in SZ patients			
Cluster 1	Area hOc2 (V2)	Perception. vision. Perception. vision. motion	Saccades
Cluster 2	L MCC	Action. inhibition Cognition Social cognition Emotion	Reward, go/no-go
Cluster 3	R precuneus	Cognition. social cognition Cognition. reasoning	Self-reflection, theory of mind
Cluster 4	Area hOc1 (V1)	No significant effects.	No significant effects.
Cluster 5	L insula	Cognition. language. semantics Cognition. language. speech	Word generation (covert) Recitation/repetition (overt)
Cluster 6	Area TE 1.0	No significant effects.	No significant effects.
Cluster 7	R insula	Action. execution. speech	Delayed match to sample Recitation/repetition (overt)
Cluster 8	R ACC	Perception. somesthesis. pain	No significant effects.
Cluster 9	L Rolandic operculum	No significant effects.	No significant effects.
Cluster 10	Area TE 1.2	No significant effects.	No significant effects.
Cluster 11	L PMF gyrus	Perception. somesthesis Perception. somesthesis. pain Action. execution	Grasping, saccades, visuospatial attention, pain monitor/discrimination, recitation/repetition (overt)

Functional characterization of the clusters retrieved from our main analyses. We used a data-driven approach to assign functional labels to clusters. In brief, this approach is based on BrainMap, a functional imaging data base, that provides functional annotations for given MNI coordinates (80, 81). We here report the functional labels that were assigned to the clusters from our main analyses above chance.

V1, visual area 1; V2, visual area 2; L, left; R, right; MCC, midcingulate cortex; ACC, anterior cingulate cortex; STL, superior temporal lobe, PMF gyrus, posterior–medial frontal gyrus.

**Figure 3:**
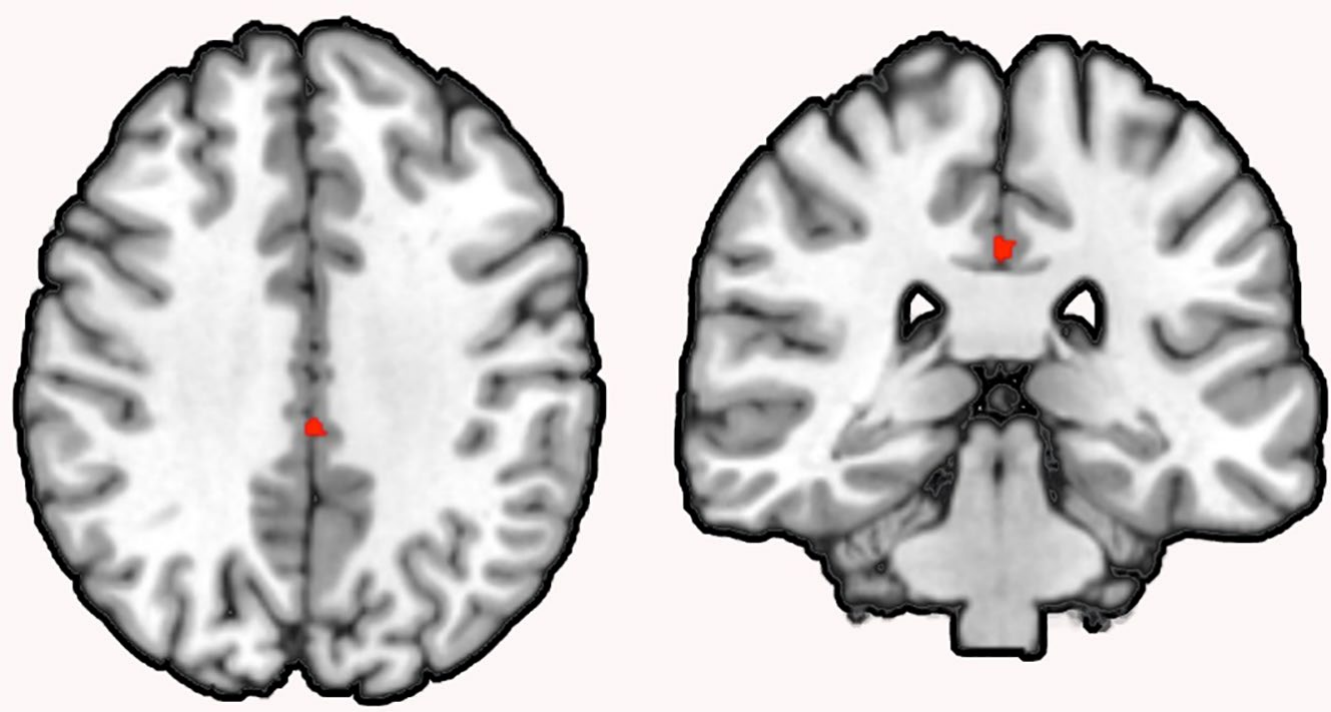
Conjunction analysis of correlation and group comparison analysis. *Note:* Conjunction analysis revealed an overlap (*k* = 60) between the NGF serum level to GMV correlation and the GMV group comparison in the left MCC in schizophrenia patients (coronal view, *p* < .001, cluster-level threshold *p* < .05, FWE-corrected, *k* > 60).

We did not find any increase in GMV in schizophrenia patients compared to healthy controls.

Subsequent conjunction analysis revealed an overlap (k = 60) between the NGF serum level to GMV correlation and the GMV group comparison in the left MCC in schizophrenia patients.

## Discussion

In this study, we found evidence for a correlation between NGF serum levels and GMV in the left prefrontal lobe, the left MCC, and the brainstem in schizophrenia patients. To the best of our knowledge, this is the first study to investigate a putative influence of NGF levels on structural neuroanatomy in schizophrenia.

Remarkably, the GMV in the left MCC was reduced in our group comparison of schizophrenia patients and healthy controls and additionally was shown to be negatively correlated to NGF serum levels in schizophrenia patients, but not in healthy controls. A conjunction analysis identified an overlap between the results of the correlation and the group comparison analysis in the left MCC.

Reduction in cingulate gyrus volume has been found in several studies in schizophrenia patients. Significant reductions were found also in first-episode patients and were shown to be progressive in longitudinal examinations (8, 16, 87). Finding the cingulate gyrus volume additionally correlated to NGF serum levels indicates an exceptional sensitivity for structural changes and supports the hypothesis of NGF signaling playing a central role in the pathogenesis of brain structural differences in a core region of schizophrenia.

Also, the left orbital gyrus volume was found to be negatively correlated with serum NGF in the patient’s group. Volume reductions in the orbital gyrus have been reported previously in schizophrenia patients (17, 88). In these studies, the volume of the left orbital gyrus was found inversely correlated with lower socioeconomic status and longer illness duration, suggesting a relation to social functioning impairment even during the first-episode of schizophrenia (88, 89). Psychopathologically, reduced GMV in the left mid orbital gyrus has been associated with more severe positive formal thought disorders (88).

Both the MCC and orbital cortex play a decisive role in social interaction (90–93). NGF is also thought to play a mechanistic role in human social interaction (94, 95). In acute psychosocial stress, NGF in saliva was found elevated (96). Interestingly, subjects reporting lower negative emotion when confronting the stressor also showed greater NGF reactivity in saliva, suggesting that this neurotrophic response might have evolved as a resilience mechanism to protect the brain against the effects of stress hormones release. These findings suggest that both salivary NGF reactivity and recovery are markers of psychiatric health (96). A recent study reported an association between functional impairment and serum NGF levels in mental disorders. NGF levels were negatively associated with social and financial autonomy and cognition (97). Given the findings of our functional decoding, MCC and orbital cortex are plausible regions mediating these functions, as being associated with emotion, cognition as well as reward-related tasks. Finding a negative correlation between the GMV in the MCC and orbital cortex with NGF serum levels can indicate an increased vulnerability for neurostructural alternations, reflecting in difficulties in social interaction and functioning (98).

In schizophrenia patients, serum NGF levels were found to be positively correlated with GMV in the pons. Functional characterization has revealed that the pontine region is linked to tasks involving pain monitoring and pain discrimination. Abnormal pain sensitivity in schizophrenia was previously reported in clinical and experimental studies, showing that schizophrenia patients appear to have diminished pain perception (99–101).

NGF, as a key regulator of nociceptive pain and an important mediator of the initiation and maintenance of different pain stages (102, 103), has an important role in pain perception. NGF antagonists have been investigated in the treatment of nociceptive and neuropathic pain conditions (102–104). Thus, the pontine cluster that was positively correlated with NGF serum levels might be a neuroanatomical correlate of NGF-mediated altered pain sensitivity in schizophrenia patients. Finding a positive correlation of serum NGF to a neuroanatomical area involved in pain management may conversely suggest an influence of altered, more precisely in terms of reduced, NGF serum levels in diminished pain perception that can be observed in schizophrenia patients.

The observation that the correlations between NGF and brain structure were exclusively present in schizophrenia patients suggests an altered effect of NGF signaling in the brains of schizophrenia patients compared to controls. There is mounting evidence for this hypothesis (50, 58, 70, 105), hinting at NGF signaling acting *via* different mechanisms in schizophrenia. Changes due to altered signaling mechanisms might be intensified through *per se* decreased NGF levels in patients. Another possibility may be a dysfunctional NGF signaling cascade, e.g., because of a higher burden of genetic lesions in patients (56).

As NGF is mainly involved in the maintenance of neurons and neuronal circuits, a positive correlation between NGF serum levels and GMV would appear as most proximate hypothesis. Two aspects, however, challenge such a straightforward model. First, while NGF might indeed lead to decreased efficiency of neural networks, this does not have to manifest necessarily in brain atrophy detectable by MRI. Contrariwise, (failed) compensatory reactions might lead to increased regional GMV. Second, as the molecular architecture of the human brain arguably differs across regions (106), reactions of complex neural tissues toward reduced NGF levels do not necessarily have to be uniform across all brain regions. While these considerations might provide some explanation for detection of both positive and negative correlations between GMV and NGF levels across brain regions, we, though, have to acknowledge that such interpretations remain highly speculative. Future studies will have to corroborate these findings and follow up further on their mechanistic underpinnings.

In our cross-sectional comparison to healthy controls, we found a significant reduction of GMV in schizophrenia patients in the visual system, the limbic system, parietal lobe, bilateral insula, primary auditory cortex, and frontal lobe. Structural alterations particularly for fronto-temporal and limbic gray matter in schizophrenia patients have been described before (5, 8, 17, 26). Functional characterization revealed a prominent functional conjunction between clusters showing reductions in GMV with social cognition, reasoning, action execution, and language-related processes, which are domains related to core symptoms of schizophrenia (107–114). Previous study hints that some of these regions are related to severity of symptoms, so, for instance, there are indications that reduced GMV in the primary visual areas is associated with increased severity of visual hallucinations in schizophrenia patients (115).

Both BMI (69) as well as antipsychotics (24) have been shown to exert an influence on NGF serum levels, and have also been discussed as being correlated with brain structural changes (21, 116). For our sample, we did not find a correlation between these two parameters and NGF levels. Consequently, we would assume that our correlation analyses were not driven by these confounds. However, future studies on larger collectives will have to investigate these relationships further, to corroborate our findings.

### Nerve Growth Factor Pathways in Schizophrenia

Neurotrophins, such as NGF, are known to pass the blood–brain barrier in both directions (46, 49). Kale et al. demonstrated a close relationship between CNS and peripheral NGF levels in healthy controls, but this correlation could not be found in drug-naive psychotic patients (50).

NGF is expressed throughout the cortex and of unique importance for maintenance and function of cholinergic neurons (117). The signaling pathways of NGF are still not fully understood. One pathway of survival, axonal growth, and differentiation of neural cells is mediated by NGF through binding to tropomyosine-related kinase (Trk)-receptor A (117, 118). NGF induces a calcium-dependent release of dopamine and glutamine (119) in a dose-dependent fashion (120) through a p75 neurotrophin receptor-dependent pathway (120, 121). Glutamate-gated ion channels as N-methyl-D-aspartate receptors (NMDARs) are widely distributed throughout the cortex (122), playing key roles in excitatory synaptic transmission and having a central role in synaptic plasticity (123).

There is a strong indication that NMDARs may play an important role in schizophrenia and psychosis (123, 124). Proven changes in the glutamatergic metabolism and transmission may potentially affect regional neuronal integrity and therefore be related to the morphological brain changes and symptoms in schizophrenia patients (122, 125). On the other hand, NMDA dysfunction may also affect the impaired dopaminergic and GABAergic neurotransmission documented in schizophrenia (122). NMDA and dopamine dysfunction are widely recognized to play a central role in disturbed neurotransmission of schizophrenia patients (126).

Different mechanisms in NGF signaling cascade, moreover enhanced through changes in NGF levels, may thereby lead to a less support on a trophic level and may in consequence contribute to changes in brain morphology (117, 127).

### Limitations

The presented study follows an exploratory approach and the results of this study should be interpreted in the context of certain limitations. Although NGF levels have shown to be stable in peripheral blood (128), a single measurement of NGF serum levels cannot reflect the influence of NGF on long-term brain structural changes.

Moreover, NGF levels in peripheral blood only partially reflect actual level and activities of NGF in the CNS, since NGF is mainly produced in the CNS, but also produced, stored, and released in several immune cells like monocytes and lymphocytes (129) and platelets (130–134). Recent evidence points to a sufficient correlation between peripheral and central nervous NGF levels, whereas this correlation was only shown in healthy controls but not in schizophrenia patients (51). A disturbed blood–brain barrier in schizophrenia patients is discussed to contribute to the development of neuromorphological changes, maybe thereby provoking the raising of NGF autoantibodies (59). However, intensified research on NGF as a proxy marker for NGF in brain tissue is needed.

Although our study showed no correlation between NGF serum level and olanzapine equivalent, NGF level has been shown to be affected by medication in other studies, predominantly by adjusting the NGF serum level to levels of healthy subjects (24, 135–140).

NGF serum levels were not symmetrically distributed in schizophrenia patients. While the small sample size did not allow to follow up on potential subgroups, this will be an important issue for future studies.

Several aspects support the actual biological relevance of our findings: first, a variety of potentially relevant covariates did not show significant correlations with NGF serum levels in the general sample or in any of the subgroups. Only for the healthy controls, we found significant correlations of NGF serum levels with age and years of education. Both covariates might have an influence on brain structure. However, it should be noted that significant correlations between NGF serum levels and brain structure were found only for schizophrenia patients and not in healthy controls. Second, none of the analyzed covariates were significantly correlated with NGF serum levels in schizophrenia patients. We would regard this as an argument that our findings were indeed associated with NGF serum levels and not driven by another covariate. Third, the affine transformation of our analysis approach will correct for overall brain volume. This should also help to correct for effects associated with age and gender. While the use of more covariates should help to further correct against biases in larger samples, we would regard these aspects as good indicators that the results reported in this pilot study were not mainly driven by covariates.

The comparatively small sample size is certainly an important limitation of our exploratory pilot study. While we certainly would like to caution the reader regarding premature conclusions drawn from our findings, we would see our results as an encouraging lead toward a relationship between NGF levels and brain structural changes in schizophrenia, and a strong urge for future studies enrolling larger samples. A larger sample size may consider covariates like sex, age, medication, or illness duration in a more solid way. Finally, a larger sample size may uncover more subtle alterations in the brain structure of schizophrenia patients and decrease the danger of false positive findings. As a follow-up to our study, analysis of the precursor protein of NGF and the interaction between the precursor and the mature form might indicate other signaling pathways and effects (117). Extension of the study concept by DTI analysis of white matter as well as functional imaging *via* fMRI may be of great interest. Moreover, a longitudinal study may be desirable to investigate the potential effect of NGF on brain structure changes in temporal course. Delineating the effects of NGF levels on early brain development would be desirable in individuals that later present with schizophrenia. Such a correlation could be investigated, e.g., in larger longitudinal cohorts of at-risk individual at younger ages.

### Conclusion

Our pilot study offered hints about the influence of serum NGF on the brain structure of several regions in schizophrenia patients, while no comparable results had been found in healthy controls. Remarkably, changes correlated with NGF serum levels manifested also in the cingulate cortex, a region that also showed volume reductions our group comparison between patients and controls and has been repeatedly implicated in the literature as morphologically altered in schizophrenia. While our sample certainly was small, we regard these findings as an encouraging indication for further research. The recruitment of larger samples will be an important goal for future studies.

## Ethics Statement

The study protocol was approved by the local ethics committee of the RWTH Aachen University. Before being enrolled in the study, written informed consent was obtained from each subject in accordance with the Declaration of Helsinki.

## Author Contributions

TN-J contributed to the conception and design of the study, and supervised data acquisition, analysis, and interpretation, as well as manuscript proofreading. LM, MS, and AN conducted informed consent. WG arranged MRI settings. KN, TW, and VK performed analysis of the MRI data. KN contributed to data acquisition, analysis, interpretation, and wrote the manuscript. CH contributed to data acquisition. TW, TA, WG, and UH contributed to manuscript proofreading.

## Funding

This work was sponsored by the foundation program “START” of the Ministry of Science and Research of the state of North Rhine-Westphalia, Germany.

## Conflict of Interest Statement

The authors declare that the research was conducted in the absence of any commercial or financial relationships that could be construed as a potential conflict of interest.

## Footnotes

1
https://www.bosterbio.com/picokine_elisa_kit_datasheet.php?sku=EK0469
