# Corrigendum: Nerve Growth Factor Serum Levels Are Associated With Regional Gray Matter Volume Differences in Schizophrenia Patients

**DOI:** 10.3389/fpsyt.2019.00908

**Published:** 2019-12-20

**Authors:** Kristina Neugebauer, Christine Hammans, Tobias Wensing, Vinod Kumar, Wolfgang Grodd, Lea Mevissen, Melanie A. Sternkopf, Ana Novakovic, Ted Abel, Ute Habel, Thomas Nickl-Jockschat

**Affiliations:** ^1^ Department of Psychiatry, Psychotherapy and Psychosomatics, Medical Faculty, RWTH Aachen University, Aachen, Germany; ^2^ Jülich-Aachen Research Alliance, Jülich, Germany; ^3^ Max-Planck-Institute for Biological Cybernetics, Tübingen, Germany; ^4^ Iowa Neuroscience Institute, University of Iowa, Iowa City, IA, United States; ^5^ Department of Psychiatry, Carver College of Medicine, University of Iowa, Iowa City, IA, United States

**Keywords:** schizophrenia, nerve growth factor, voxel-based morphometry, neuroimaging, functional decoding

In the original article, there was an error. The **Author Contributions** statement misrepresents the contributions of TN-J, KN, TW, TA, WG, and UH.

A correction has been made to **Author Contributions**:

“TN-J contributed to the conception and design of the study, and supervised data acquisition, analysis, and interpretation, as well as manuscript proofreading. LM, MS, and AN conducted informed consent. WG arranged MRI settings. KN, TW, and VK performed analysis of the MRI data. KN contributed to data acquisition, analysis, interpretation, and wrote the manuscript. CH contributed to data acquisition. TW, TA, WG, and UH contributed to manuscript proofreading.”

The authors apologize for this error and state that this does not change the scientific conclusions of the article in any way. The original article has been updated.

