# Supplementary material for: Nerve Growth Factor Serum Levels Are Associated With Regional Gray Matter Volume Differences in Schizophrenia Patients
Source: Front Psychiatry. 2019 Apr 26;10:275. doi: 10.3389/fpsyt.2019.00275 (PMC6498747; doi:10.3389/fpsyt.2019.00275)
Supplement: Supplementary file 1 [file Table_1.docx]

**11.6 Supplementary Table 1 Intra assay variations**

|  | Value 1 | Value 2 | Mean |  |  | Value 1 | Value 2 | Mean |
| --- | --- | --- | --- | --- | --- | --- | --- | --- |
| SZ 1 | 0,049 | 0,049 | 0,049 |  | HC 1 | 0,06 | not detect. | 0,06 |
| SZ 2 | 0,056 | 0,052 | 0,054 |  | SC 2 | 0,056 | not detect. | 0,056 |
| SZ 3 | 0,051 | 0,055 | 0,053 |  | HC 3 | 0,051 | 0,056 | 0,0535 |
| SZ 4 | 0,078 | 0,064 | 0,071 |  | HC 4 | 0,055 | 0,057 | 0,056 |
| SZ 5 | 0,046 | 0,05 | 0,048 |  | HC 5 | 0,063 | 0,064 | 0,0635 |
| SZ 6 | 0,053 | 0,057 | 0,055 |  | HC 6 | 0,069 | 0,072 | 0,0705 |
| SZ 7 | 0,049 | 0,055 | 0,052 |  | HC 7 | 0,055 | 0,055 | 0,055 |
| SZ 8 | 0,054 | 0,063 | 0,0585 |  | HC 8 | 0,073 | 0,07 | 0,0715 |
| SZ 9 | 0,053 | 0,051 | 0,052 |  | HC 9 | 0,054 | 0,054 | 0,054 |
| SZ 10 | 0,046 | 0,052 | 0,049 |  | HC 10 | 0,063 | 0,066 | 0,0645 |
| SZ 11 | 0,056 | 0,056 | 0,056 |  | HC 11 | 0,054 | 0,053 | 0,0535 |
| SZ 12 | 0,055 | 0,054 | 0,0545 |  | HC 12 | 0,05 | 0,053 | 0,0515 |
| SZ 13 | 0,06 | 0,062 | 0,061 |  | HC 13 | 0,054 | 0,052 | 0,053 |
| SZ 14 | 0,054 | 0,05 | 0,052 |  | HC 14 | 0,046 | 0,046 | 0,046 |
| SZ 15 | 0,05 | 0,056 | 0,053 |  | HC 15 | 0,05 | 0,079 | 0,0645 |
| SZ 16 | 0,058 | 0,054 | 0,056 |  | HC 16 | 0,052 | 0,06 | 0,056 |
| SZ 17 | 0,051 | 0,055 | 0,053 |  | HC 17 | 0,05 | 0,052 | 0,051 |
| SZ 18 | 0,059 | 0,054 | 0,0565 |  | HC 18 | 0,056 | 0,057 | 0,0565 |
|  |  |  |  |  | HC 19 | 0,055 | 0,052 | 0,0535 |
|  |  | Mean Patients | 0,05463889 pg/ml |  |  |  | Mean Controls | 0,05736842 pg/ml |
